# Supplementary material for: Automated Microsolvation for Minimum Energy Path Construction in Solution
Source: J Chem Theory Comput. 2025 May 28;21(11):5571–87. doi: 10.1021/acs.jctc.5c00245 (PMC12160001; doi:10.1021/acs.jctc.5c00245)
Supplement: Supplementary file 1 [file ct5c00245_si_001.pdf]

Supporting Information

Automated Microsolvation for Minimum Energy Path Construction in Solution

Paul L. Türtscher[1] and Markus Reiher[2,*]

Department of Chemistry and Applied Biosciences, ETH Zurich, Vladimir-Prelog-Weg 2, 8093 Zurich, Switzerland

[1]ORCID: 0000-0002-7021-5643
[2]Corresponding author; e-mail: mreiher@ethz.ch; ORCID: 0000-0002-9508-1565

1 Effect of Scaling Factor s

In the following, we discuss the effect of varying the scaling factor s (compare Eq. 1) for the methanediol formation from formaldehyde and water. The default scaling factor s for all examples shown in this work is 2.3. Here, we additionally ran our protocol on the identical solute-solvent complexes, but with scaling factors of 2.0, 2.6, 2.9, and 3.2. The overall effect on key parameters are summarized in Table S1.

s Factor	# mQM Reactions	# sQM Reactions	Av. # QM solv. molecules in mQM region	Av. mQM Runtime [min]
2.0	39	52	5(3)	15
2.3	34	50	5(2)	16
2.6	11	33	7(1)	25
2.9	8	17	15(4)	27
3.2	3	8	17(1)	33

Table S1: Effect of a varying scaling factor s on the number of reactions found at the mQM stage and the sQM stage of our protocol, as well as the average number of QM solvent molecules in the mQM region with the corresponding standard deviation and the average runtime for step 1 and step 2 of our protocol.

Based on the results in Table S1, we find the effect of s in the range between $s = 2.0$ and $s = 2.3$ to be very similar. With a higher scaling factor, $s > 2.3$, the average runtime increases due to an increase of the number of solvent molecules in the QM region for the costly QM/MM transition state optimization. This causes a decrease in the number of reactions found in step 2 of our protocol, because the transition state optimization from the corresponding transition state guess structure often fails to maximize one eigenvector with negative eigenvalue. As a consequence, the number of sQM reactions determined in step 3 of our protocol decreases as well.

In the following, we present plots summarizing the results obtained with scaling factors of 2.0, 2.6, 2.9, and 3.2. For $s = 2.3$, the corresponding plots can be either found in Section 5.1.1 in the main manuscript or in Section 2.1 in the SI. For groups with more than two data points, box plots are added where the whiskers of the boxes indicate the minimum and maximum values. The box borders depict the second and third quartile, and the line in a box denotes the median of the energies of the group. The dashed red line indicates the free energy of activation of $125.6\,\mathrm{kJ\,mol^{-1}}$ only considering continuum solvation. The yellow dashed line indicates the experimental value of the free energy of activation of $67.1\,\mathrm{kJ\,mol^{-1}}$.[1]

1.1 Supplementary Plots for Scaling Factor of 2.0

Comparing the results with a scaling factor s of 2.0 to those obtained with our default value of $s = 2.3$, the number of reactions with more than three active solvent molecules is smaller. As we are interested in finding these rarer TSs as well, a scaling factor of 2.3 is more appropriate.

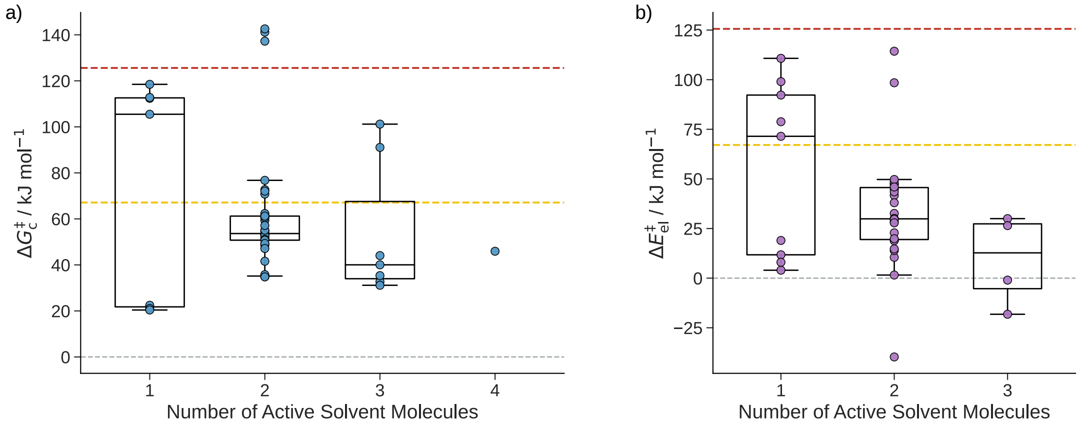

Figure S1: **a)** sQM free energies of activation $\Delta G^{\ddagger}_{c}$ of the reaction of formaldehyde with water to form methanediol with $s = 2.0$, grouped by the number of active solvent molecules. **b)** mQM/MM energies of activation of the hydrolysis of formaldehyde with $s = 2.0$, grouped by the number of active solvent molecules.

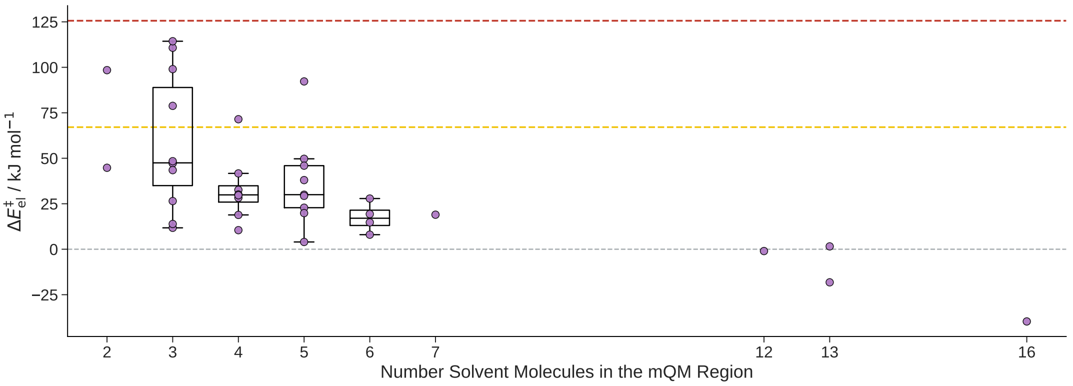

Figure S2: mQM/MM energies of activation of the methanediol formation from formaldehyde with $s = 2.0$ grouped by the number of solvent molecules in the mQM region.

1.2 Supplementary Plots for Scaling Factor of 2.6

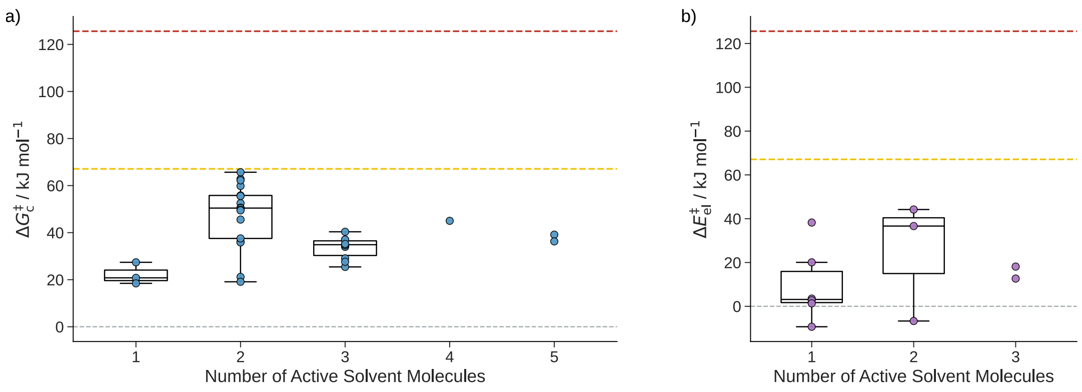

Figure S3: **a)** sQM free energies of activation $\Delta G^{\ddagger}_{c}$ of the reaction of formaldehyde with water to form methanediol with $s = 2.6$, grouped by the number of active solvent molecules. **b)** mQM/MM energies of activation of the hydrolysis of formaldehyde with $s = 2.6$, grouped by the number of active solvent molecules.

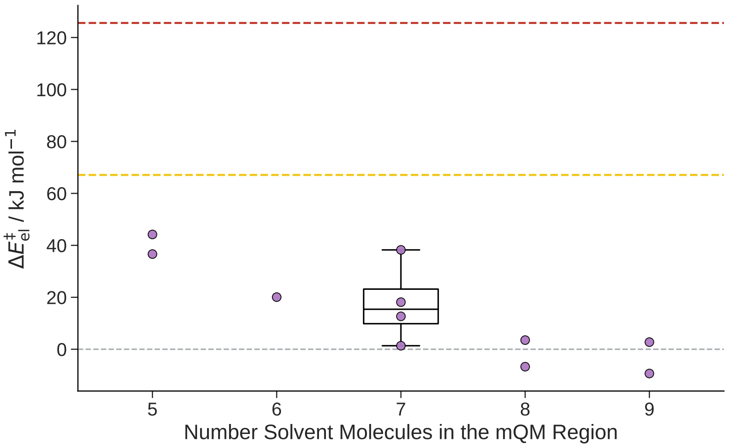

Figure S4: mQM/MM energies of activation of the methanediol formation from formaldehyde with $s = 2.6$ grouped by the number of solvent molecules in the mQM region.

1.3 Supplementary Plots for Scaling Factor of 2.9

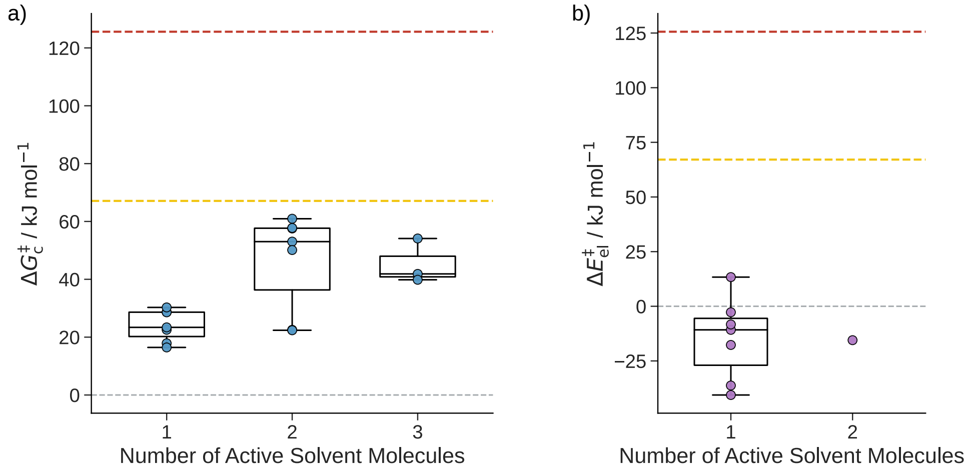

Figure S5: **a)** sQM free energies of activation $\Delta G^{\ddagger}_{\mathrm{c}}$ of the reaction of formaldehyde with water to form methanediol with $s = 2.9$, grouped by the number of active solvent molecules. **b)** mQM/MM energies of activation of the hydrolysis of formaldehyde with $s = 2.9$, grouped by the number of active solvent molecules.

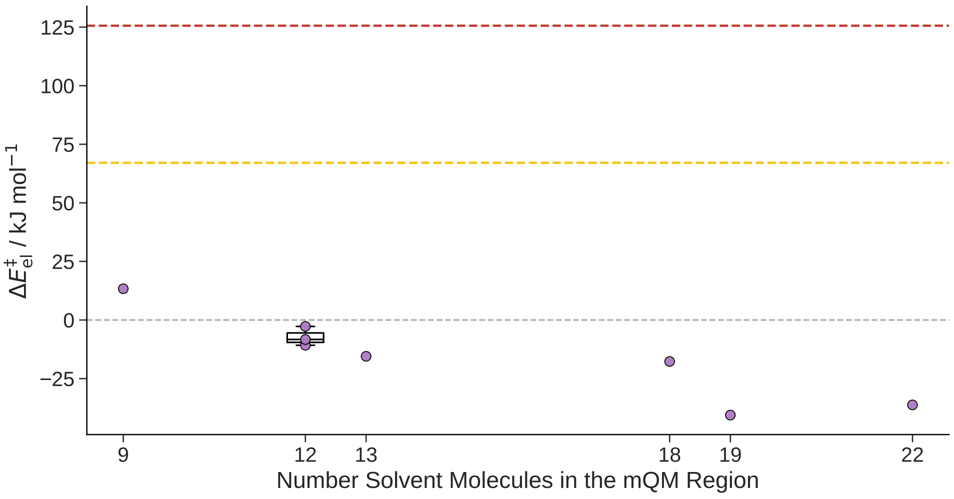

Figure S6: mQM/MM energies of activation of the methanediol formation from formaldehyde with $s = 2.9$ grouped by the number of solvent molecules in the mQM region.

1.4 Supplementary Plots for Scaling Factor of 3.2

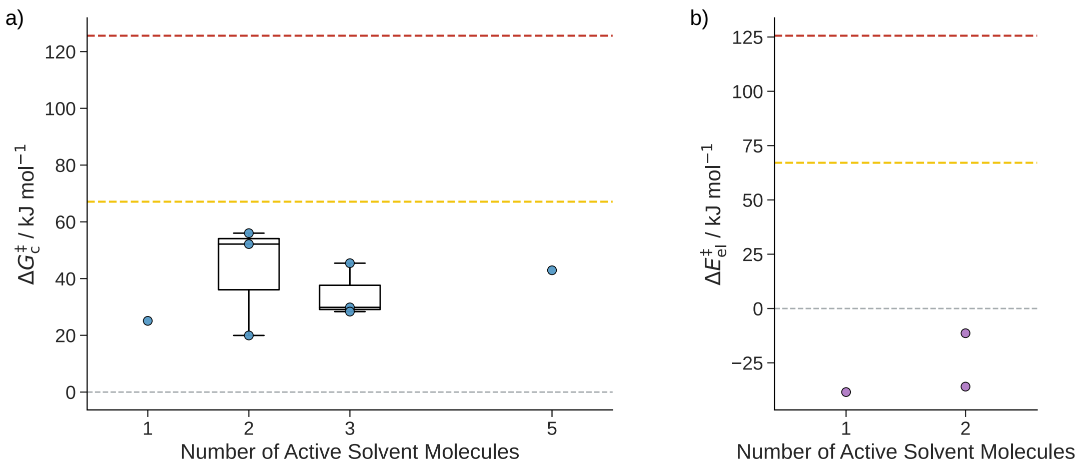

Figure S7: **a)** sQM free energies of activation $\Delta G^{\ddagger}_{\mathrm{c}}$ of the reaction of formaldehyde with water to form methanediol with $s = 3.2$, grouped by the number of active solvent molecules. **b)** mQM/MM energies of activation of the hydrolysis of formaldehyde with $s = 3.2$, grouped by the number of active solvent molecules.

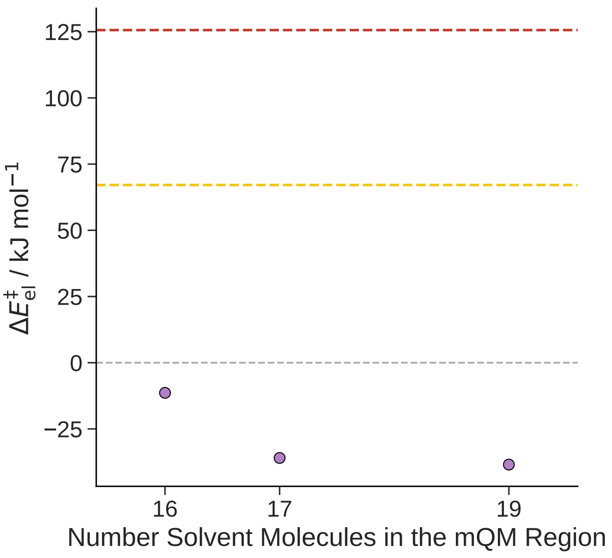

Figure S8: mQM/MM energies of activation of the methanediol formation from formaldehyde with $s = 3.2$ grouped by the number of solvent molecules in the mQM region.

2 Supplementary Plots of Activation Energies

Here, we provide additional plots of the presented activation energies where they are grouped by the total number of solvent molecules, solvent molecules in the mQM regions, and total number of solvents in the sQM region.

2.1 Methanediol Formation from Formaldehyde

All plots presented contain the following features. For groups with more than two data points, box plots are added where the whiskers of the boxes indicate the minimum and maximum values. The box borders depict the second and third quartile, and the line in a box denotes the median of the energies of the group. The dashed red line indicates the free energy of activation of 125.6 kJ mol^{-1} only considering continuum solvation. The yellow dashed line indicates the experimental reference value of the free energy of activation of 67.1 kJ mol^{-1}.[1]

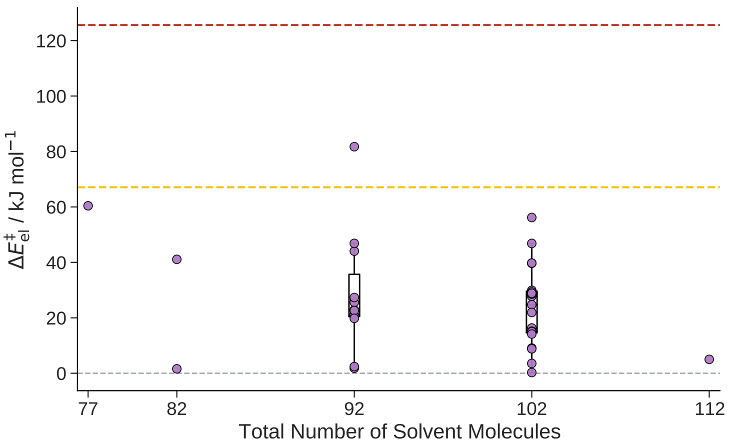

Figure S9: mQM/MM energies of activation of the methanediol formation from formaldehyde grouped by the number of total solvent molecules in the full mQM/MM system.

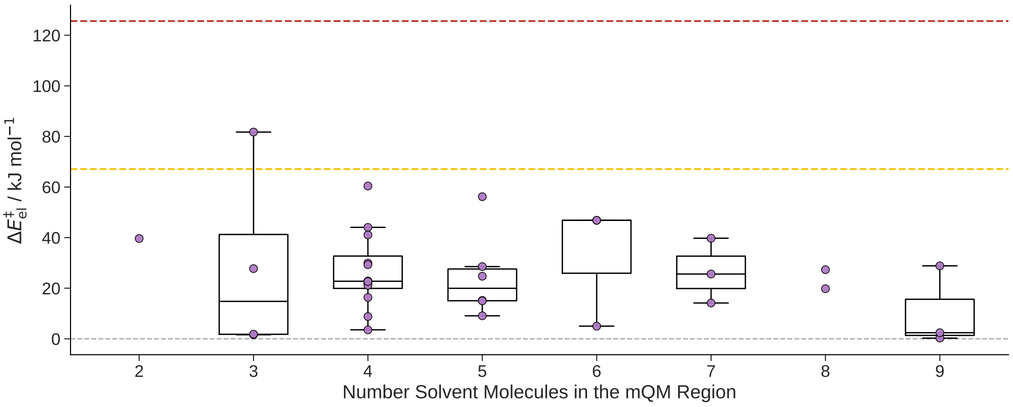

Figure S10: mQM/MM energies of activation of the methanediol formation from formaldehyde grouped by the number of solvent molecules in the mQM region.

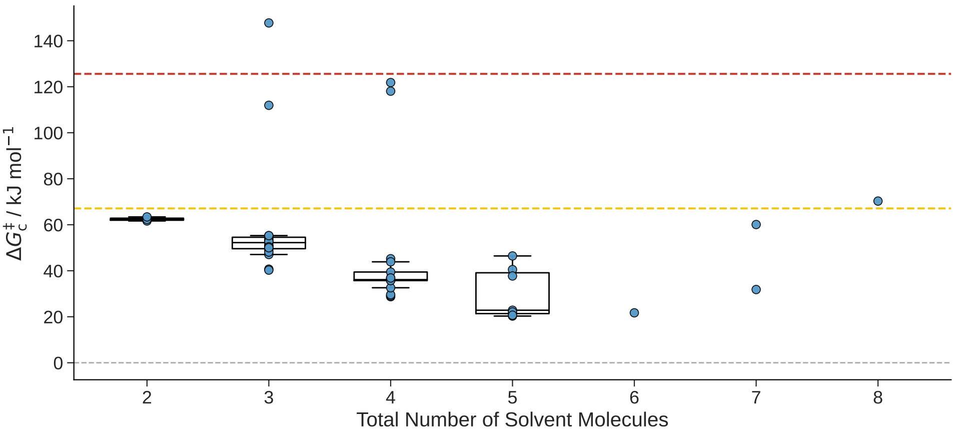

Figure S11: sQM free energies of activation of the methanediol formation from formaldehyde grouped by the number of total solvent molecules.

2.2 Chlorination of Phenol

All plots presented contain the following features. For groups with more than two data points, box plots are added where the whiskers of the boxes indicate the minimum and maximum values, disregarding outliers. The box borders depict the second and third quartile, and the line in each box denotes the median of the energies of a group. The yellow dashed line indicates the experimental reference value of the free energy of activation of 75.6(19) kJ mol^{-1}.[2]

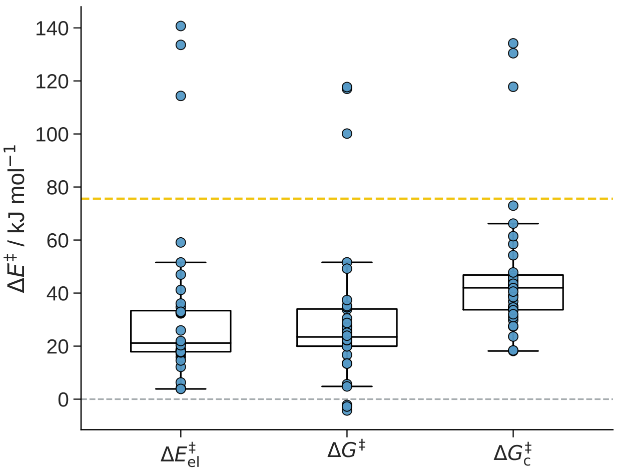

Figure S12: Comparison of sQM $\Delta E^{\ddagger}_{el}$ with free energy corrected energies of activation of the σ-complex formation from phenol in water.

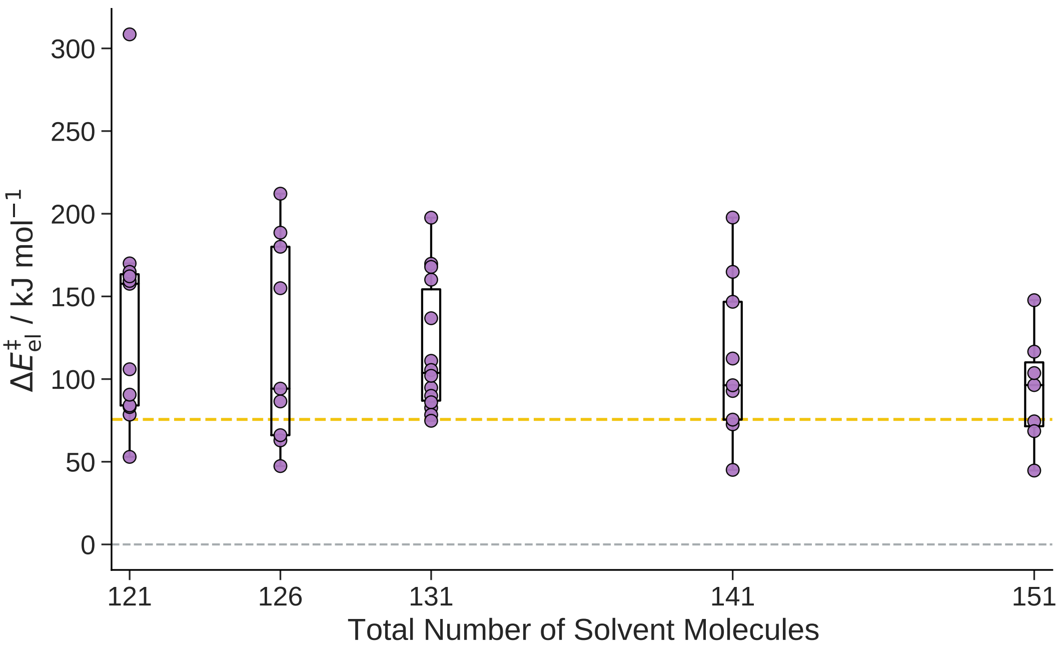

Figure S13: mQM/MM energies of activation of the σ-complex formation from phenol in water grouped by the number of total solvent molecules in the full mQM/MM system.

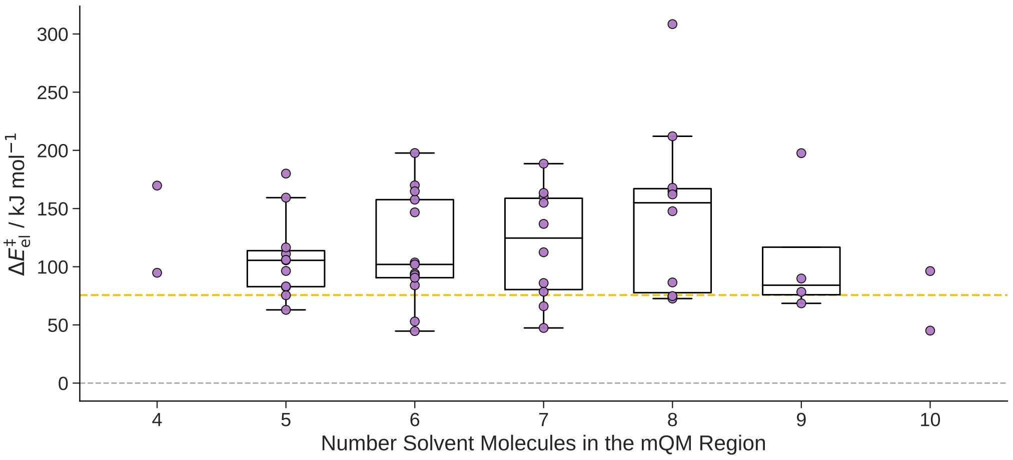

Figure S14: mQM/MM energies of activation of the σ-complex formation from phenol in water grouped by the number of solvent molecules in the mQM region.

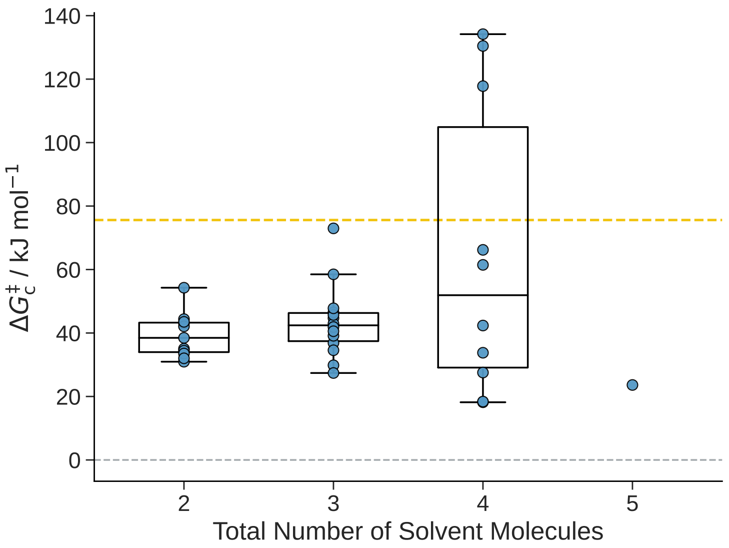

Figure S15: sQM free energies of activation of the σ-complex formation from phenol in water grouped by the number of total solvent molecules.

2.3 Hydration of CO_2

All plots presented contain the following features. For groups with more than two data points, box plots are added where the whiskers of the boxes indicate the minimum and maximum values, disregarding outliers. The box borders depict the second and third quartile, and the line in each box denotes the median of the energies of a group. The dashed red line indicates the free energy of activation of 169.2 kJ mol^{-1}

and 169.8 kJ mol^{-1} only considering continuum solvation for pure water and a 1:1 mixture of water and methanol, respectively. The yellow dashed line indicates the experimental reference value of the free energy of activation of 91.2 kJ mol^{-1}.[3]

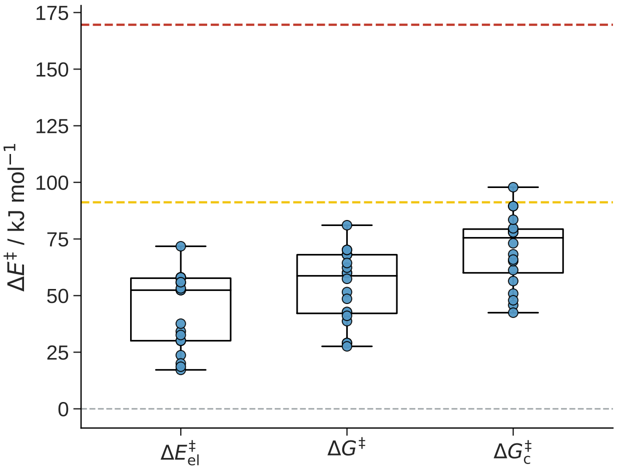

Figure S16: Comparison of sQM energies of activation of the hydration of CO_2.

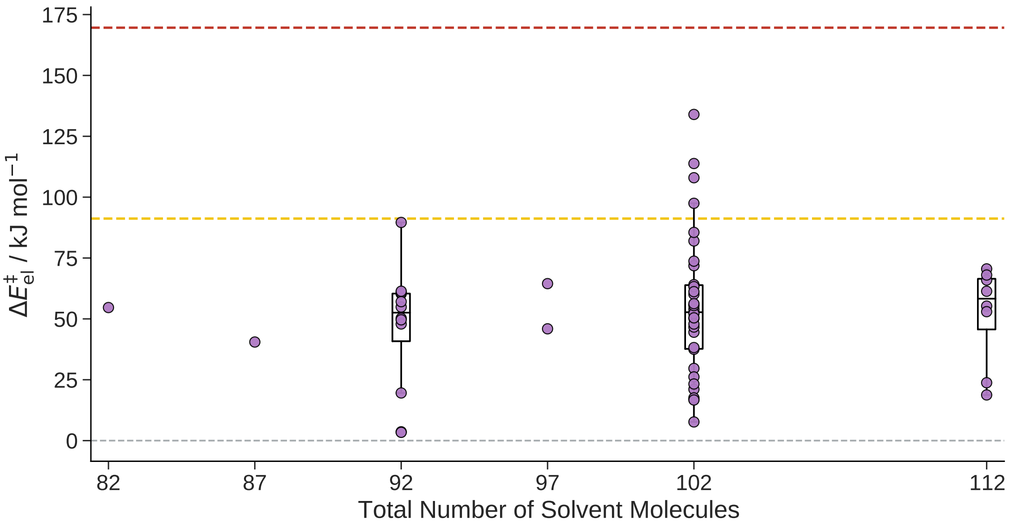

Figure S17: mQM/MM energies of activation of the hydration of CO_2 grouped by the number of total solvent molecules in the full mQM/MM system.

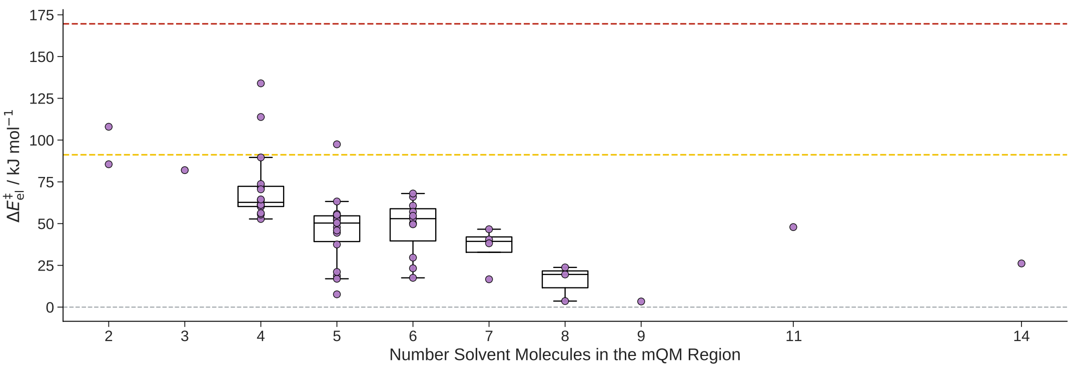

Figure S18: mQM/MM energies of activation of the hydration of CO_2 grouped by the number of total solvent molecules in the mQM region.

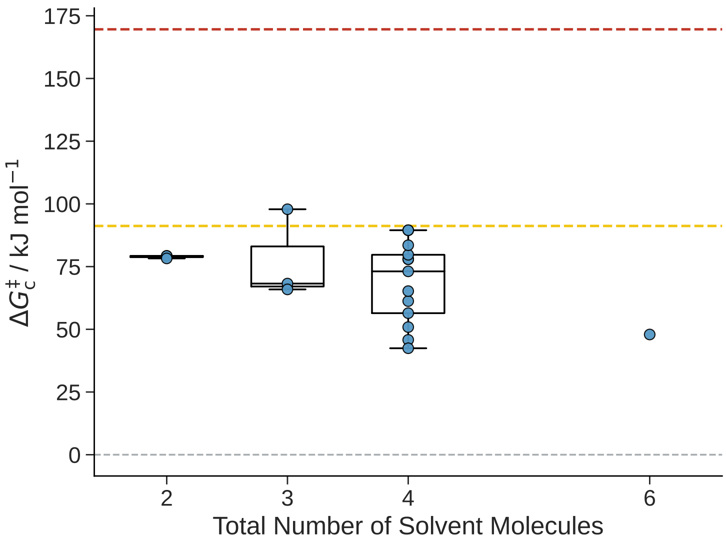

Figure S19: sQM free energies of activation of the hydration of CO_2 grouped by the number of total solvent molecules.

2.3.1 Hydration of CO_2 in a water/methanol mixture

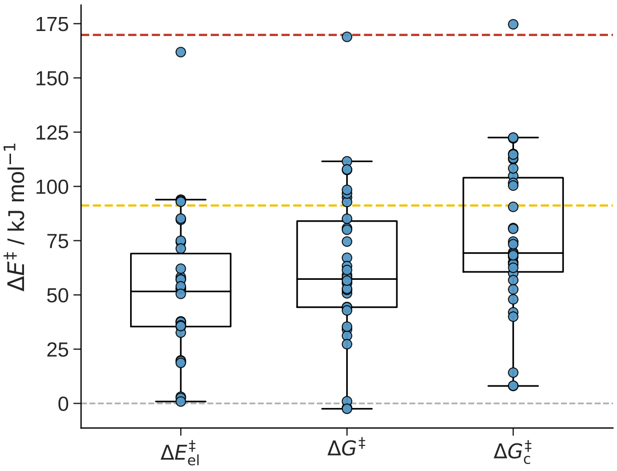

Figure S20: Comparison of sQM energies of activation of the hydration of CO_2 in a water/methanol mixture.

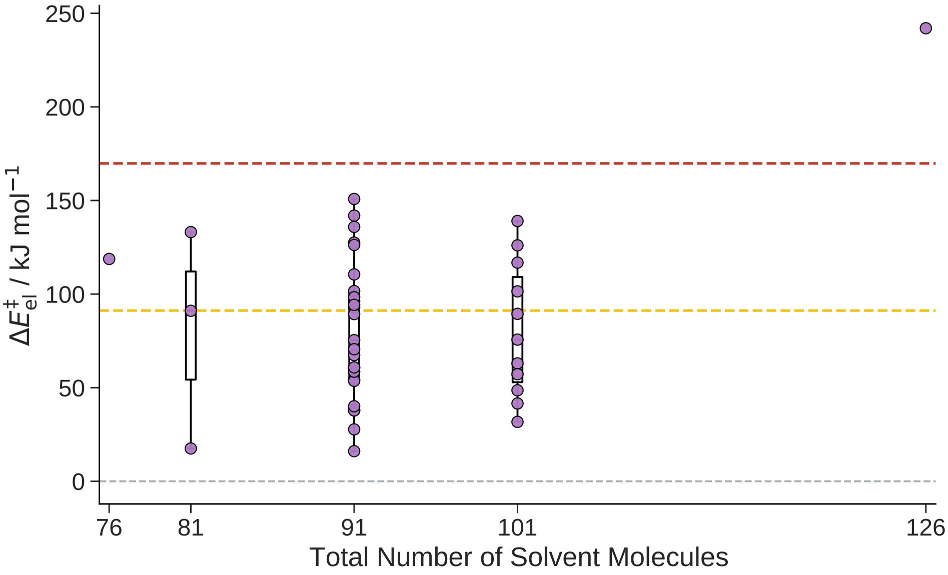

Figure S21: mQM/MM energies of activation of the hydration of CO_2 in a water/methanol mixture grouped by the number of total solvent molecules in the full mQM/MM system.

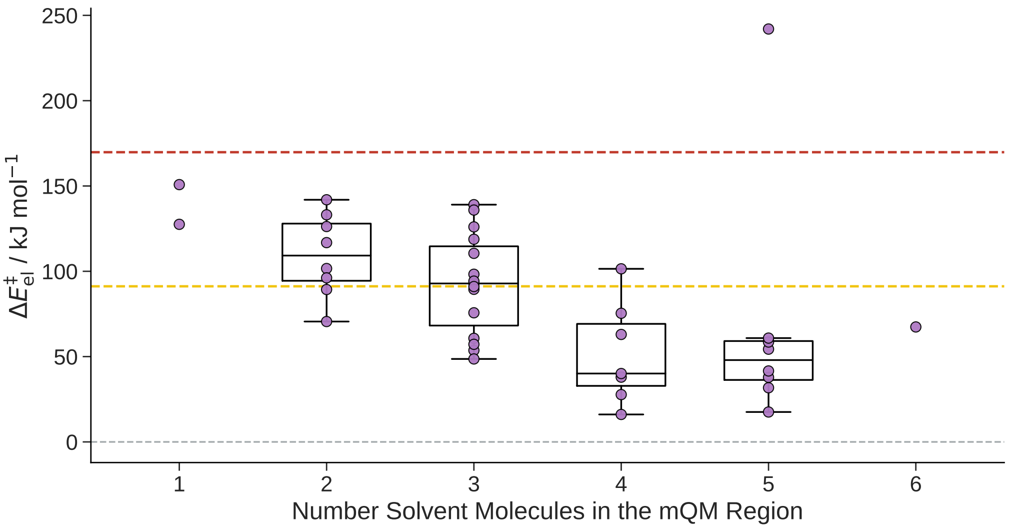

Figure S22: mQM/MM energies of activation of the hydration of CO_2 in a water/methanol mixture grouped by the number of total solvent molecules in the mQM region.

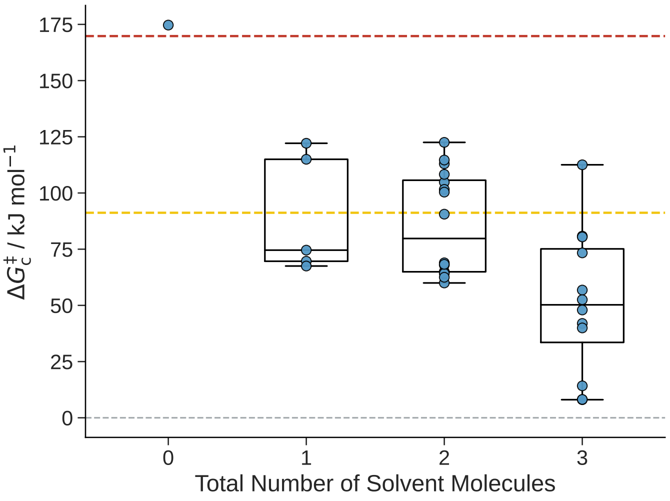

Figure S23: sQM free energies of activation of the hydration of CO_2 in a water/methanol mixture grouped by the number of total solvent molecules.

3 Damped Vibrational Entropy of Activation

Here, we follow the work of Grimme and of Conquest et al.[4,5] The damped vibrational entropy is determined as a weighted sum of the vibrational entropy and the corresponding entropy of a one dimensional rigid rotor $S_{\text{rot,1D}}$. The moment of inertia μ of the rigid rotor is derived from the frequency ν_i of the normal mode i,

$$S_{\text{vib,damped}} = R \sum_i w(\nu_i) S_{\text{vib}} + (1 - w(\nu_i))\, S_{\text{rot,1D}}. \tag{1}$$

Here, $w(\nu_i)$ corresponds to a damping factor and is defined as

$$w(\nu_i) = \frac{1}{1 + (\nu_{\text{th}}/\nu_i)^4} \tag{2}$$

where the low frequency threshold ν_{th} in this work is set to $100\,\text{cm}^{-1}$. The rotational entropy of the one dimensional rotor is defined with

$$S_{\text{rot,1D}} = R\left[\frac{1}{2} + \frac{1}{2}\ln\left(\frac{8\pi^3 k_{\text{B}} T}{h^2}\mu'\right)\right] \tag{3}$$

where μ' is the reduced moment of inertia derived from the corresponding normal mode frequency ν_i and the average molecular moment of inertia with

$$\mu' = \frac{\mu\,(I_a I_b I_c)^{\frac{1}{3}}}{\mu + (I_a I_b I_c)^{\frac{1}{3}}} \tag{4}$$

$$\mu = \frac{h}{8\pi^2 \nu_i}. \tag{5}$$

For calculating the free energy of activation, $\Delta G^{\ddagger}_{\text{c,d}}$, considering the damped contributions of the vibrational entropy and the cavity entropy, we exploit the following equation

$$\Delta G^{\ddagger}_{\text{c,d}} = \Delta H^{\ddagger} - T\left(\Delta S^{\ddagger}_{\text{rot}} + \Delta S^{\ddagger}_{\text{vib,damped}} + \Delta S^{\ddagger}_{\text{c}}\right). \tag{6}$$

.

3.1 Activation Energies with Damped Entropies of Activation

All plots presented contain the following features. For groups with more than two data points, box plots are added where the whiskers of the boxes indicate the minimum and maximum values. The box borders depict the second and third quartile, and the line in a box denotes the median of the energies of the group.

3.1.1 Methanediol Formation from Formaldehyde

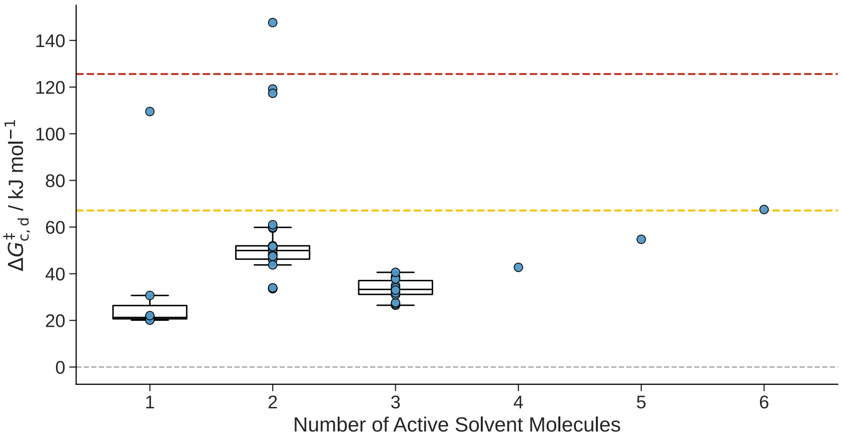

Figure S24: sQM free energies of activation $\Delta G^{\ddagger}_{\mathrm{c,d}}$ of the reaction of formaldehyde with water to form methanediol grouped by the number of active solvent molecules. The dashed red line indicates the free energy of activation of 125.6 kJ mol^{-1} considering solely continuum solvation. The yellow dashed line indicates the experimental reference value of the free energy of activation of 67.1 kJ mol^{-1}.[1]

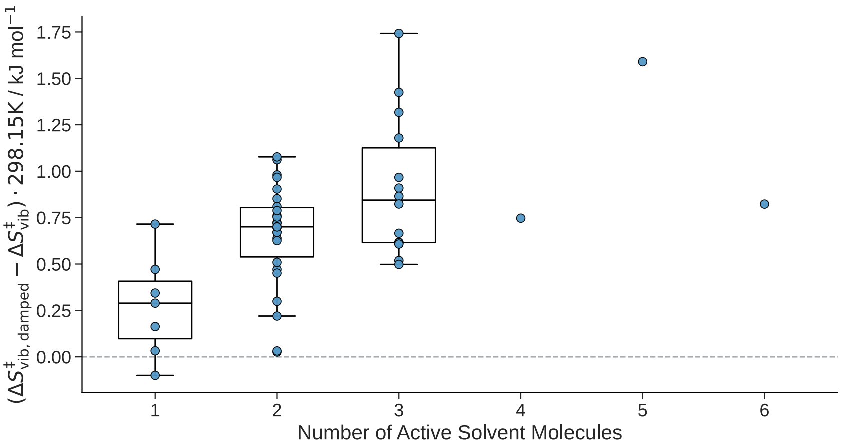

Figure S25: Differences of the damped entropies of activation $\Delta S^{\ddagger}_{\mathrm{vib,\,damped}}$ and the undamped entropies of activation $\Delta S^{\ddagger}_{\mathrm{vib}}$ multiplied by 298.15 K of the reaction of formaldehyde with water to form methanediol grouped by the number of active solvent molecules.

3.1.2 Chlorination of Phenol

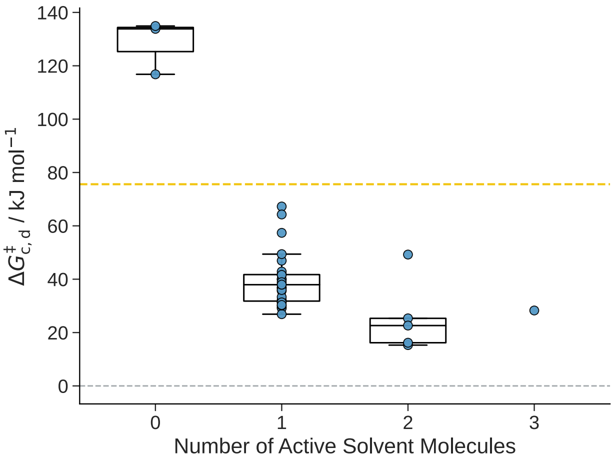

Figure S26: sQM free energies of activation $\Delta G^{\ddagger}_{\mathrm{c,d}}$ for the σ-complex formation from phenol in water sorted according to the number of active solvent molecules found in different configurations. The yellow dashed line indicates the experimental reference value of the free energy of activation of the chlorination of phenol 75.6(19) kJ mol^{-1}.[2]

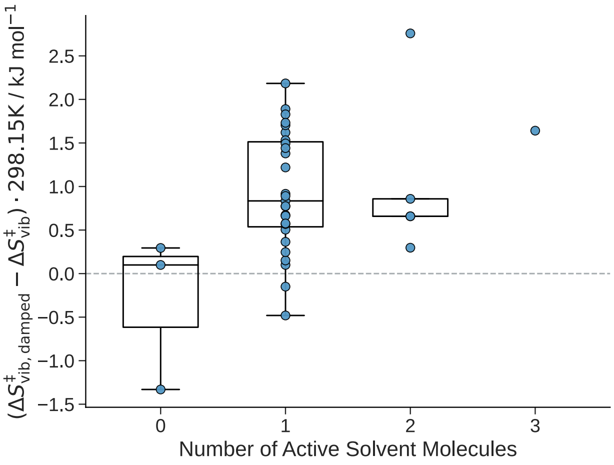

Figure S27: Differences of the damped entropies of activation, $\Delta S^{\ddagger}_{\mathrm{vib,\,damped}}$, and the undamped entropies of activation, $\Delta S^{\ddagger}_{\mathrm{vib}}$, multiplied by 298.15 K for the σ-complex formation from phenol in water sorted according to the number of active solvent molecules found in different configurations.

3.1.3 Hydration of CO_2

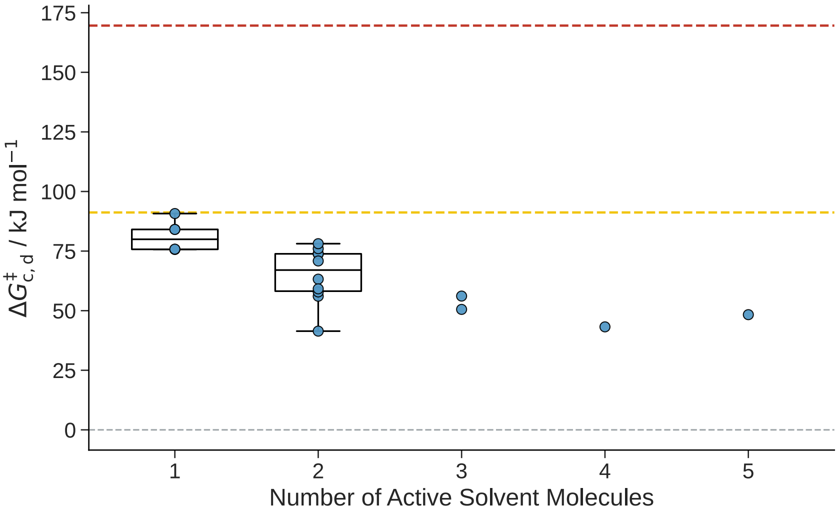

Figure S28: sQM free energies of activation $\Delta G^{\ddagger}_{\mathrm{c,d}}$ of the hydration of CO_2 grouped by the number of active solvent molecules. The dashed red line indicates the free energy of activation of 169.6 kJ mol^{-1} only considering continuum solvation. The yellow dashed line indicates the experimental reference value of the free energy of activation of 90.2 kJ mol^{-1}.[3]

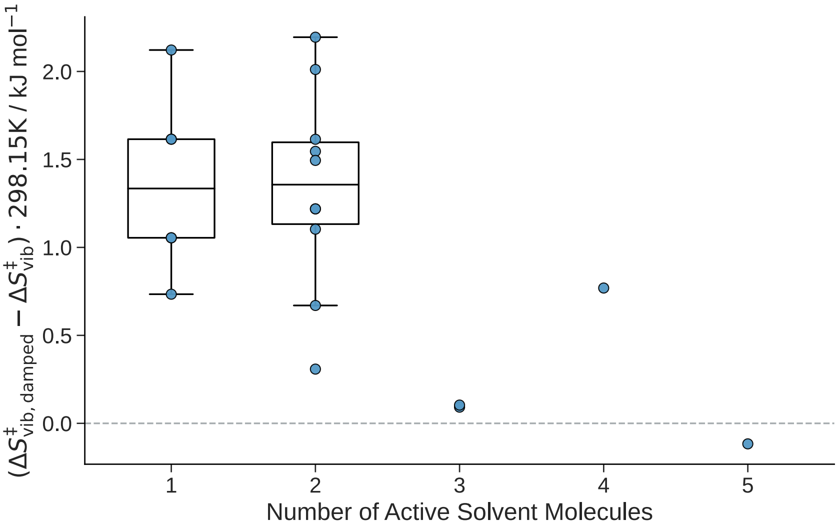

Figure S29: Differences of the damped entropies of activation, $\Delta S^{\ddagger}_{\mathrm{vib,\ damped}}$, and the undamped entropies of activation, $\Delta S^{\ddagger}_{\mathrm{vib}}$, multiplied by 298.15 K of the hydration of CO_2 grouped by the number of active solvent molecules.

3.1.4 Hydration of CO_2 in a water/methanol mixture

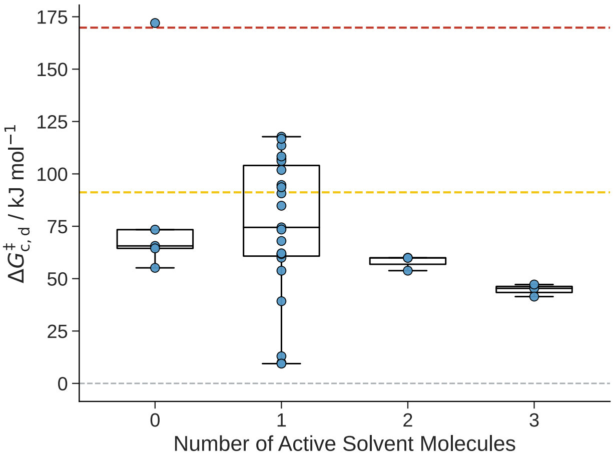

Figure S30: sQM free energies of activation $\Delta G^{\ddagger}_{\mathrm{c,d}}$ of the hydration of CO_2 in a water/methanol mixture grouped by the number of active solvent molecules. The dashed red line indicates the free energy of activation of 169.8 kJ mol^{-1} only considering continuum solvation. The yellow dashed line indicates the experimental reference value of the free energy of activation in pure water of 90.2 kJ mol^{-1}.[3]

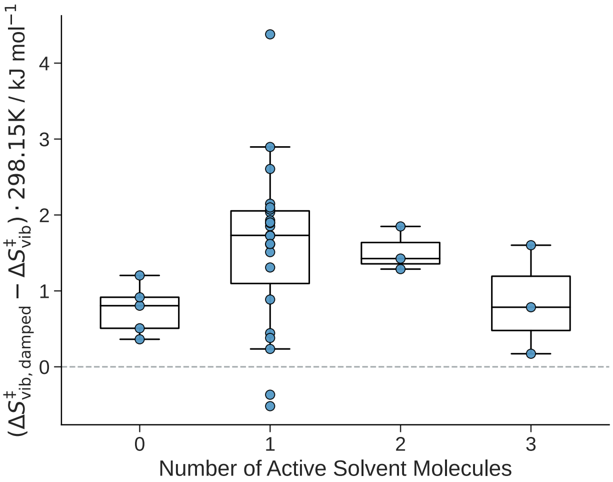

Figure S31: Differences of the damped entropies of activation, $\Delta S^{\ddagger}_{\mathrm{vib,\,damped}}$, and the undamped entropies of activation, $\Delta S^{\ddagger}_{\mathrm{vib}}$, multiplied by 298.15 K of the hydration of CO_2 in a water/methanol mixture grouped by the number of active solvent molecules.

References

[1] Winkelman, J. G. M.; Voorwinde, O. K.; Ottens, M.; Beenackers, A. A. C. M.; Janssen, L. P. B. M. Kinetics and Chemical Equilibrium of the Hydration of Formaldehyde. *Chem. Eng. Sci.* **2002**, *57*, 4067–4076.

[2] Gallard, H.; von Gunten, U. Chlorination of Phenols: Kinetics and Formation of Chloroform. *Environ. Sci. Technol.* **2002**, *36*, 884–890.

[3] Wang, X.; Conway, W.; Burns, R.; McCann, N.; Maeder, M. Comprehensive Study of the Hydration and Dehydration Reactions of Carbon Dioxide in Aqueous Solution. *J. Phys. Chem. A* **2010**, *114*, 1734–1740.

[4] Grimme, S. Supramolecular Binding Thermodynamics by Dispersion-Corrected Density Functional Theory. *Chem. Eur. J.* **2012**, *18*, 9955–9964.

[5] Conquest, O. J.; Roman, T.; Marianov, A.; Kochubei, A.; Jiang, Y.; Stampfl, C. Calculating Entropies of Large Molecules in Aqueous Phase. *J. Chem. Theory Comput.* **2021**, *17*, 7753–7771.
